# State-Selected Reactivity of Carbon Dioxide Cations (${\text{CO}}_{2}^{+}$) With Methane

**DOI:** 10.3389/fchem.2019.00537

**Published:** 2019-08-02

**Authors:** Daniela Ascenzi, Claire Romanzin, Allan Lopes, Paolo Tosi, Jan Žabka, Miroslav Polášek, Christopher J. Shaffer, Christian Alcaraz

**Affiliations:** ^1^Department of Physics, University of Trento, Trento, Italy; ^2^Laboratoire de Chimie Physique, Bât. 350, UMR 8000, CNRS-Univ. Paris-Sud and Paris Saclay, Centre Universitaire Paris-Sud, Orsay, France; ^3^Synchrotron SOLEIL, L'Orme des Merisiers, Saint-Aubin—BP 48, Gif-sur-Yvette, France; ^4^J. Heyrovský Institute of Physical Chemistry of the Czech Academy of Sciences, Prague, Czechia; ^5^Institute of Organic Chemistry and Biochemistry of the Czech Academy of Sciences, Prague, Czechia

**Keywords:** vibrational excitation, plasma, astrochemistry, Mars atmosphere, synchrotron radiation, ion-molecule reaction, CO_2_ dissociation

## Abstract

The reactivity of ${\text{CO}}_{2}^{+}$ with CD_4_ has been experimentally investigated for its relevance in the chemistry of plasmas used for the conversion of CO_2_ in carbon-neutral fuels. Non-equilibrium plasmas are currently explored for their capability to activate very stable molecules (such as methane and carbon dioxide) and initiate a series of reactions involving highly reactive species (e.g., radicals and ions) eventually leading to the desired products. Energy, in the form of kinetic or internal excitation of reagents, influences chemical reactions. However, putting the same amount of energy in a different form may affect the reactivity differently. In this paper, we investigate the reaction of ${\text{CO}}_{2}^{+}$ with methane by changing either the kinetic energy of ${\text{CO}}_{2}^{+}$ or its vibrational excitation. The experiments were performed by a guided ion beam apparatus coupled to synchrotron radiation in the VUV energy range to produce vibrationally excited ions. We find that the reactivity depends on the reagent collision energy, but not so much on the vibrational excitation of ${\text{CO}}_{2}^{+}$. Concerning the product branching ratios (${\text{CD}}_{4}^{+}$/${\text{CD}}_{3}^{+}$/DOCO^+^) there is substantial disagreement among the values reported in the literature. We find that the dominant channel is the production of ${\text{CD}}_{4}^{+}$, followed by DOCO^+^ and ${\text{CD}}_{3}^{+}$, as a minor endothermic channel.

## Introduction

The chemistry of the ${\text{CO}}_{2}^{+}$ cation attracts much attention because of the presence of this ion in planetary atmospheres (with particular reference to the Earth and Mars Matta et al., 2013; Tenewitz et al., 2018 as well as in laboratory plasmas for energetic and environmental applications Snoeckx and Bogaerts, 2017). In the latter field, the efficient conversion of greenhouse gases into value-added chemicals is a central topic in current research on renewable and sustainable energies (Wang et al., 2017). In particular, the hydrogenation of CO_2_ by technologies based on green electricity allows both the storage of renewable energy in value-added compounds and recycling CO_2_, thus paving the way to decarbonise the energy system. Non-thermal plasmas have been explored for their capability to activate very stable molecules with the potential of achieving a higher energy efficiency compared to purely thermal processes (Scapinello et al., 2016; Martini et al., 2018). To improve the performances and to control the outcome of plasma-based processes, insight into the physical and chemical mechanisms at play is desired. According to a chemical kinetic model of the plasma-based dry reforming (Snoeckx et al., 2013), a key role is played by the reaction of ${\text{CO}}_{2}^{+}$ with CH_4_. However, as described below, there is considerable uncertainty on the branching ratio, so that a reinvestigation of the reaction is desirable. Also, because vibrationally excited levels of ${\text{CO}}_{2}^{+}$ can be populated in plasmas, this study aims at investigating the effect of the vibrational excitation of the ${\text{CO}}_{2}^{+}$ cation on the reaction with CH_4_.

Energy, in the form of kinetic or internal motion of the reagents, is the driving force of chemical reactions. However, putting the same amount of energy in a different form (i.e., translational, vibrational, rotational or electronic energy) may affect the reactivity differently. For ion-molecule reactions, some state-selected experiments have shown that for endothermic charge-transfer (CT) processes, vibrational excitation is more effective than translational energy in driving the reactions (Viggiano and Morris, 1996). However, in other cases, the effect of vibrational excitation is more varied (see for example Candori et al., 2003; Boyle et al., 2011; Chang et al., 2012; Bell and Anderson, 2013a,b; Bell et al., 2014 and reference therein).

The effect of the internal excitation of ${\text{CO}}_{2}^{+}$ in reactions with small molecules has been addressed in previous studies. It was found that the vibrational excitation of ${\text{CO}}_{2}^{+}$ increases the reactivity with O_2_ and NO (Alge et al., 1981; Durup-Ferguson et al., 1983; Derai et al., 1985; Ferguson et al., 1992; Nicolas et al., 2002), while it decreases the rate coefficient for the reaction with H_2_ (Albritton, 1979; Borodi et al., 2009). However, no previous studies exist in which the ${\text{CO}}_{2}^{+}$ cation is generated with a precise amount of internal energy (i.e., state-selection of a specific vibrational state) and reacted with CH_4_.

## Previous Studies of the ${\text{CO}}_{2}^{+}$ + CH_4_ Reaction

The reaction of ${\text{CO}}_{2}^{+}$ with methane in the gas phase has been studied by several groups, with the earliest experimental results dating back to the late 60s (Anicich, 2003). Rate constant and product branching ratio measurements were made using drift techniques, either flow drift tubes (FDT) (Rakshit and Warneck, 1980; Durup-Ferguson et al., 1983) or selected ion flow tube (SIFT) (Smith et al., 1978; Copp et al., 1982), ion cyclotron resonance (ICR) techniques (Huntress et al., 1980) and ion beam methods (Tsuji et al., 1994). Earlier determinations where done using high-pressure mass spectrometry (HPMS) (Harrison and Myher, 1967; Chong and Franklin, 1971; Kasper and Franklin, 1972) and electron space charge traps (SCT) (Ryan and Harland, 1974). The most relevant results are summarized in Table 1.

**Table 1:** Summary of existing experimental determinations of rate constants and branching ratios for the reaction of ${\text{CO}}_{2}^{+}$ with CH_4_.

	**Product branching ratios**				
***k* (cm^3^·molecule^−1^·s^−1^)^a^**	**${\text{CH}}_{4}^{+}$**	**HOCO^**+**^**	**Others**	**Method**	**References**
(9.60 ± 3.8)× 10^−10^	0.28 ± 0.01	0.72 ± 0.01		Beam	Tsuji et al., 1994
(1.0 ± 0.3)× 10^−9^	1.0			FDT	Durup-Ferguson et al., 1983
(1.1 ± 0.2)× 10^−9^	0.5 ± 0.1	0.5 ± 0.1		SIFT	Copp et al., 1982
(1.0 ± 0.1)× 10^−9^	0.25	0.75		ICR	Huntress et al., 1980
(9.00 ± 1.8)× 10^−10^		1.00		DT	Rakshit and Warneck, 1980
1.0× 10^−9^	0.60	0.40		SIFT	Smith et al., 1978
(1.15 ± 0.1)× 10^−9^	0.30	0.70		SCT	Ryan and Harland, 1974
2.31× 10^−9^		>0.99	HCO^+^ <0.01	HPMS	Kasper and Franklin, 1972
1.2× 10^−9^		1.00^b^		MS	Harrison and Myher, 1967

a *Rate constants at thermal energy*.

b *Reaction with CD_4_ to give CO_2_D^+^ exclusively*.

It has been shown that the reaction proceeds at thermal energy with a rate constant close to the Langevin collision rate constant *k*_*L*_ = 1.1 × 10^−9^ cm^3^·molecule^−1^·s^−1^ (Durup-Ferguson et al., 1983). There is fair agreement (within the experimental errors) among the total rate constants measured at thermal energies (in the range 280–340 K for the data reported in Table 1), with the exception of the HPMS study by Kasper and Franklin (1972), that gives a rate constant value more than a factor two higher than the others. The values for the branching ratios are quite scattered, with HOCO^+^ being dominant in all studies except (Durup-Ferguson et al., 1983), where the CT is the only observed channel.

## Materials and Methods

The experiments have been performed using the CERISES apparatus, an associated experiment to the SOLEIL synchrotron radiation facility. Since the set-up was described in details previously (see Alcaraz et al., 2004; Cunha de Miranda et al., 2015), only the most relevant details will be given here. CERISES is a guided ion beam tandem mass spectrometer composed of two octopoles located between two quadrupole mass spectrometers in a Q1-O1-O2-Q2 configuration that permits investigation of bi-molecular reactions of mass-selected ions. By measuring the yields of parent- and product-ions, absolute reaction cross sections, branching ratios and product velocity distributions as a function of the collision energy are derived.

Vibrational state selection of ${\text{CO}}_{2}^{+}$ is performed via the Threshold Photoelectron Photoion Coincidence (TPEPICO) method (Baer and Guyon, 1986) using the ion source of CERISES and the DESIRS beamline. The undulator based DESIRS beamline (Nahon et al., 2012) provides tunable radiation in the vacuum ultraviolet (VUV) range from about 5 eV to 40 eV. Photons at the desired wavelength are selected and scanned simultaneously with the undulator peak energy by a normal incidence monochromator equipped with a low dispersion uncoated SiC grating (200 grooves/mm) optimized to provide photon flux in the 10^12^ photon/s to 10^13^ photon/s range with an energy resolution down to 1 meV in the 5 eV to 20 eV range. In the present experiments, the photon energies (*E*_*phot*_) required to produce the parent ion by photoionisation are in the range 13.7–18.3 eV. Depending on the operation mode, the monochromator slits were set in the range 25 to 400 μm, corresponding, at these photon energies, to a resolution of 3 to 44 meV.

In the TPEPICO mode, the ${\text{CO}}_{2}^{+}$ ions are extracted in coincidence with threshold photoelectrons. Threshold photoelectrons are filtered first through geometrical discrimination of energetic photoelectrons by using a small extraction field of ≈ 1 V/cm and an extraction hole of 2 mm in diameter. Further time discrimination of energetic photoelectrons is made possible by recording the photoelectron arrival time on the detector and setting a time gate of 10 ns corresponding to the arrival time of threshold photoelectrons. The overall resolution of threshold electrons is about 25 meV. The source pressure and VUV flux are set to limit the false coincidence (FC) rate in the order of 10%. The FC are measured for each TPEPICO point for parent and product ions by replacing the true photoelectron signal by an arbitrary trigger. The FC contribution is then subtracted from the ion count signal. Some measurements have been carried out in the DC mode, i.e., without state-selection but with parent ions in a distribution of excitation that can vary with the photon energy.

**Figure 1:**
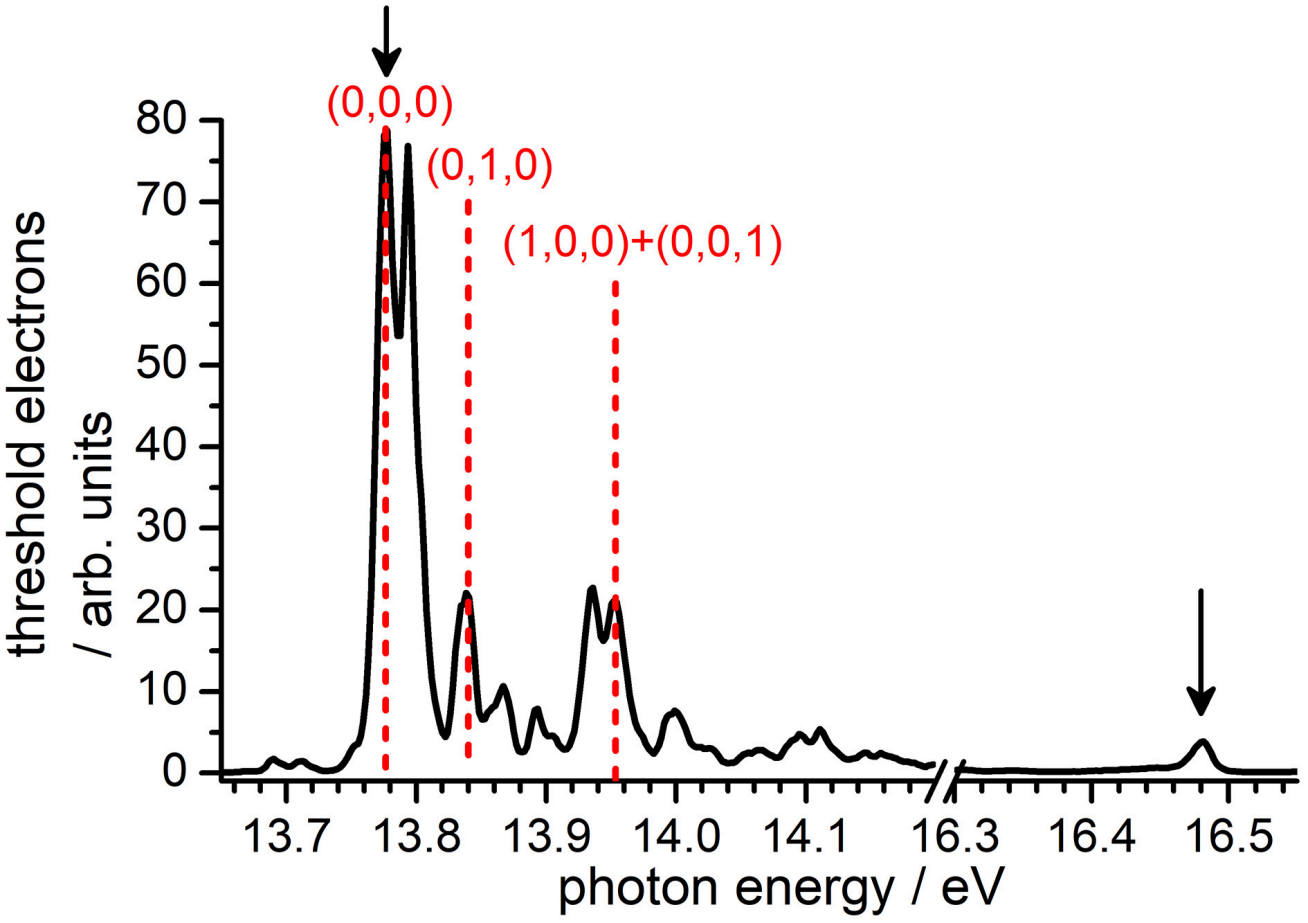
TPEPICO spectrum of CO_2_ recorded using the CERISES set-up at the DESIRS beamline of the SOLEIL synchrotron. Black solid arrows indicate the photon energies (*E*_*phot*_) at which reactivity studies in the DC mode have been performed, while red dashed lines indicate the energies chosen for experiments in the coincidence mode, i.e., with selective internal excitation of the ${\text{CO}}_{2}^{+}$ cation (see section Results and Discussion).

Prior to the reactivity experiment, the threshold photoelectron spectrum of CO_2_ has been measured using the CERISES set up in the 13.7–18.3 eV photon energy range corresponding to the ${\text{X}}^{2}\Pi_{\text{g}}$(3/2, 1/2) ground state (I.E. = 13.778 eV), the first excited state A ^2^Π _u_(3/2, 1/2) with I.E. = 17.313 eV, up to the beginning of the B^2^$\overset{+}{\underset{\text{u}}{\sum }}$ state of the ion (I.E. = 18.076 eV). For high-resolution VUV TPES spectra of CO_2_ with the complete assignment of the spectral features to specific internal modes of the ${\text{CO}}_{2}^{+}$ ion, the reader is referred to the papers by Baer and Guyon (1986); Merkt et al. (1993); Liu et al. (2000a,b). In Figure 1 we report our measured TPES spectrum in the VUV region where reactivity experiments have been performed. The red dashed lines indicate the photon energies chosen for the production of state-selected ${\text{CO}}_{2}^{+}$ in the TPEPICO mode, while the black arrows point to the photon energies at which reactivity studies have been performed in the DC mode (without state-selection).

Following the assignment of Liu et al. (2000a), the photoelectron band at *E*_*phot*_ = 13.78 eV corresponds to the transition ${\text{CO}}_{2}^{+}$(0,0,0) ${\text{X}}^{2}\Pi_{\text{g},3/2}$ ← CO_2_(0,0,0) ${\text{X}}^{1}\Sigma_{\text{g}}^{+}$, hence producing the ${\text{CO}}_{2}^{+}$ cation in its electronic ground state with no vibrational excitation, hereafter indicated as (0,0,0). The band at *E*_*phot*_ = 13.84 eV corresponds to one of the four vibronic components of the transition ${\text{CO}}_{2}^{+}$(0,1,0) ${\text{X}}^{2}\Pi_{\text{g}}$ ← CO_2_(0,0,0) X^1^$\overset{+}{\underset{\text{g}}{\sum }}$, more precisely either the Δ25/2 or ^2^Σ^+^ component that we cannot distinguish at our limited resolution (see Liu et al., 2000a paper for the attribution of the four components), thus generating the ${\text{CO}}_{2}^{+}$ cation in its electronic ground state with one quantum of vibrational excitation in the bending ν_2_ mode, hereafter indicated as (0,1,0). The band at *E*_*phot*_ = 13.95 eV corresponds to the overlap of two transitions, namely ${\text{CO}}_{2}^{+}$(1,0,0) X ∏2g,1/2 ← CO_2_(0,0,0) X Σ1g+ and ${\text{CO}}_{2}^{+}$(0,0,1) X ∏2g,3/2 ← CO_2_(0,0,0) ${\text{X}}^{1}\Sigma_{\text{g}}^{+}$, and therefore leads to ${\text{CO}}_{2}^{+}$ in the X state with one quantum of vibrational excitation in either the symmetric (ν_1_) or the antisymmetric (ν_3_) stretching vibration, hereafter indicated as (1,0,0) + (0,0,1).

A sufficient number of ion counts on the threshold electron signal have been observed to perform reactivity experiments with the state-selected cations at the three photon energies (red lines in Figure 1) corresponding to the vibronic bands giving ${\text{CO}}_{2}^{+}$ with either no or low vibrational excitation. The intensity of other vibronic bands corresponding to higher internal excitation of ${\text{CO}}_{2}^{+}$ was not sufficient to study reactivity in coincidence. For this reason, we decided to perform some measurements not in the coincidence mode, i.e., without pure state-selection but with parent ions in a distribution of excitation that can vary with the photon energy. Two photon-energies were chosen: at *E*_*phot*_ = 13.48 eV, corresponding to the ${\text{CO}}_{2}^{+}$(0,0,0) ${\text{X}}^{2}\Pi_{\text{g},3/2}$ ← CO_2_(0,0,0) ${\text{X}}^{1}\Sigma_{\text{g}}^{+}$ transition, the ${\text{CO}}_{2}^{+}$ cation will be produced with no vibrational excitation; at *E*_*phot*_ = 16.48 eV, corresponding to a strong resonant autoionization transition via Rydberg states converging to the $\tilde{B}$ state of ${\text{CO}}_{2}^{+}$, the latter will be produced in the X electronic state but with a broad distribution of internal energies, hence in a mixture of low and high vibrational excitation (see Baer and Guyon, 1986 for details).

Deuterated methane (CD_4_) was used for the reactivity study, to avoid partial mass overlap between the strong parent ion peak at *m/z* 44 and the one due to the product of the H-atom transfer process at *m/z* 45. CD_4_ pressure in the scattering cell was kept in the range 1–2 × 10^−4^ mbar throughout the experiments. Some considerations on possible isotope effects arising when CD_4_ is replaced by CH_4_ are addressed in the Conclusions.

## Results and Discussion

### Results in the DC Mode

As already mentioned in the previous section, in the DC mode we have measured absolute values of the cross sections as a function of the collision energy *E*_*CM*_ at two selected photon energies: *E*_*phot*_ = 13.78 eV (no vibrational excitation of the ${\text{CO}}_{2}^{+}$ cation) and *E*_*phot*_ = 16.48 eV (some excitation to high vibrational levels). The main reaction products observed are due to the CT and deuterium-atom-transfer channels (1) and (2), respectively:

(1) $${\text{CO}}_{\text{2}}^{\text{+}}{\text{+CD}}_{\text{4}}\to {\text{CO}}_{\text{2}}{\text{+CD}}_{\text{4}}^{\text{+}}$$

(2) $$\to {\text{DCO}}_{\text{2}}^{\text{+}}{\text{+CD}}_{\text{3}}$$

Also, small amounts of ${\text{CD}}_{3}^{+}$, and minimal amounts of CD_3_${\text{CO}}_{2}^{+}$ are detected and attributed to the following channels (3), (4) and (5):

(3) $${\text{CO}}_{\text{2}}^{\text{+}}{\text{+CD}}_{\text{4}}\to {\text{CD}}_{\text{3}}^{\text{+}}\text{+DOCO}$$

(4) $$\to {\text{CD}}_{\text{3}}^{\text{+}}{\text{+D+CO}}_{\text{2}}$$

(5) $$\to {\text{CD}}_{\text{3}}{\text{CO}}_{\text{2}}^{\text{+}}\text{+D}$$

Using literature values (taken from NIST Chemistry Webbook^1^, with the exception of some products as specified in the following) for the standard enthalpies of formations (Δ_f_*H*°) of reagents and products we can assess that both channels (1) and (2) are exothermic, while channels (3) and (4) are endothermic. In particular, the CT channel (1) has a reaction enthalpy Δ_r_*H*° = −1.17 eV, while Δ_r_*H*° for (2) is equal to −1.26 eV, assuming that HOCO^+^ has the structure of the hydroxyformyl cation (Holmes et al., 2006). The production of ${\text{CD}}_{3}^{+}$ can derive either from D^−^ transfer process (3) or from dissociative CT (4). In the former case, the DOCO radical might be formed in association with the methyl cation, and the overall process is calculated to be endothermic by about 0.56 eV, using the heat of formation of HOCO as reported in Francisco et al. (2010). In the latter case, the methyl cation derives from dissociation of the CT product and the process is endothermic by about 0.61 eV.

The CD_3_${\text{CO}}_{2}^{+}$ formation can be due to D loss from the ion-molecule adduct ${\text{CO}}_{2}^{+}$-CD_4_ (reaction 5). We can attempt to estimate the reaction enthalpy of (5) assuming that CD_3_${\text{CO}}_{2}^{+}$ has the structure of the methoxycarbonyl cation [an average value for its heat of formation Δ_f_*H*°(CH_3_OCO^+^) is 5.57±0.19 eV, as reported in Holmes et al., 2006] to get Δ_r_*H*° = −1.10 eV. We note in passing that the adduct ${\text{CO}}_{2}^{+}$-CD_4_ was not observed, as expected, as the CD_4_ pressure in the scattering cell used throughout the experiments (about 2 × 10^−4^ mbar) was too low to allow for secondary collisions for its stabilization.

**Figure 2:**
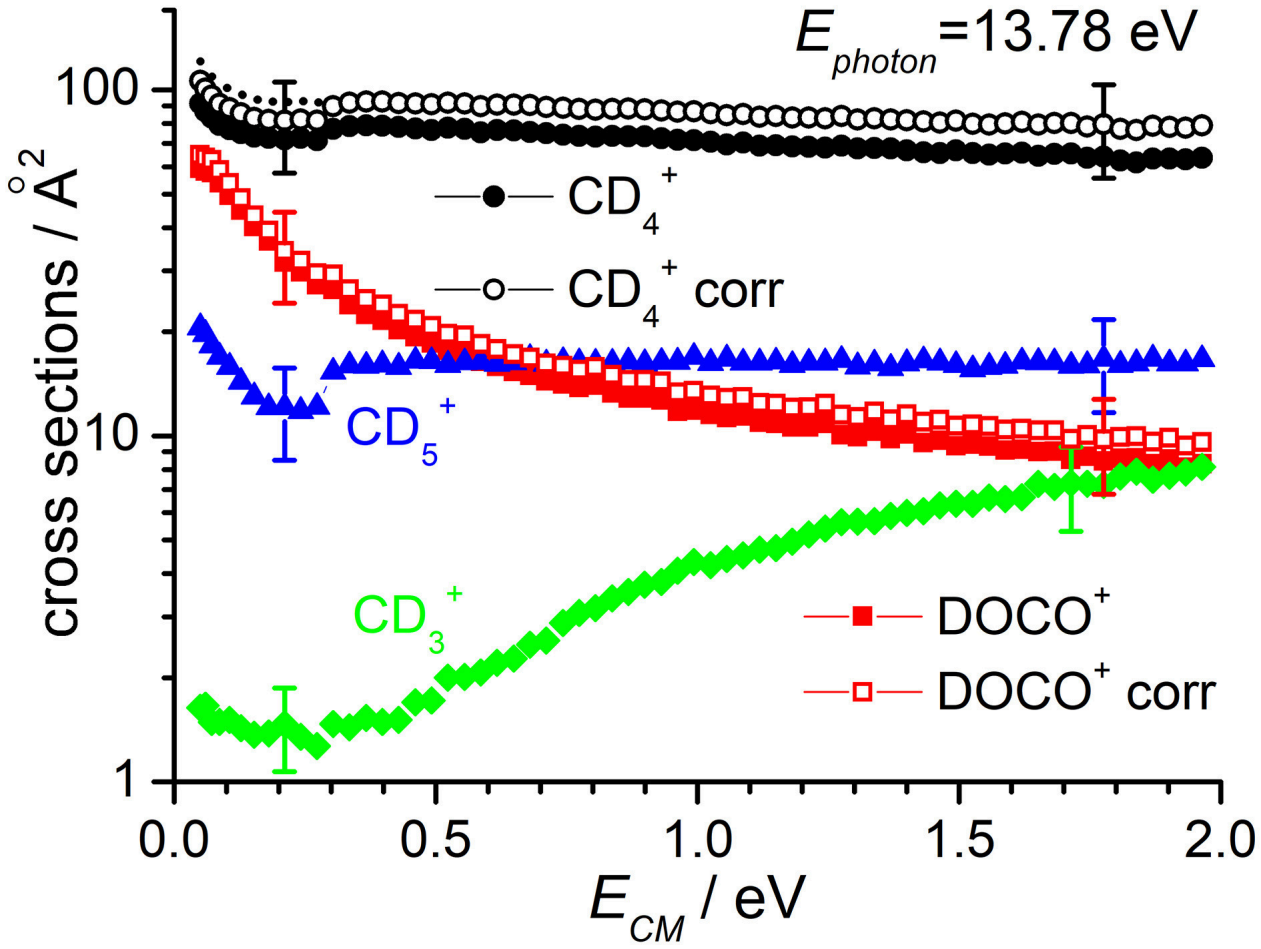
Reactive cross sections for products ${\text{CD}}_{4}^{+}$ (uncorrected, black circles), DOCO^+^ (uncorrected, red squares), ${\text{CD}}_{3}^{+}$ (green diamonds), and ${\text{CD}}_{5}^{+}$ (blue triangles) measured as a function of the collision energy (*E*_*CM*_) in the DC mode at a photon energy *E*_*phot*_ = 13.78 eV. The open black circles and open red squares are the ${\text{CD}}_{4}^{+}$ and DOCO^+^ cross sections corrected to include the contribution of secondary reactions leading to ${\text{CD}}_{5}^{+}$. The dotted line represents the ${\text{CD}}_{4}^{+}$ cross sections corrected for the instrumental effect due to decreased collection efficiency at low *E*_*CM*_ (see text for details). Error bars on all the data are about 30%: for the sake of clarity only two error bars are reported, at arbitrarily chosen low and high collision energy values.

**Figure 3:**
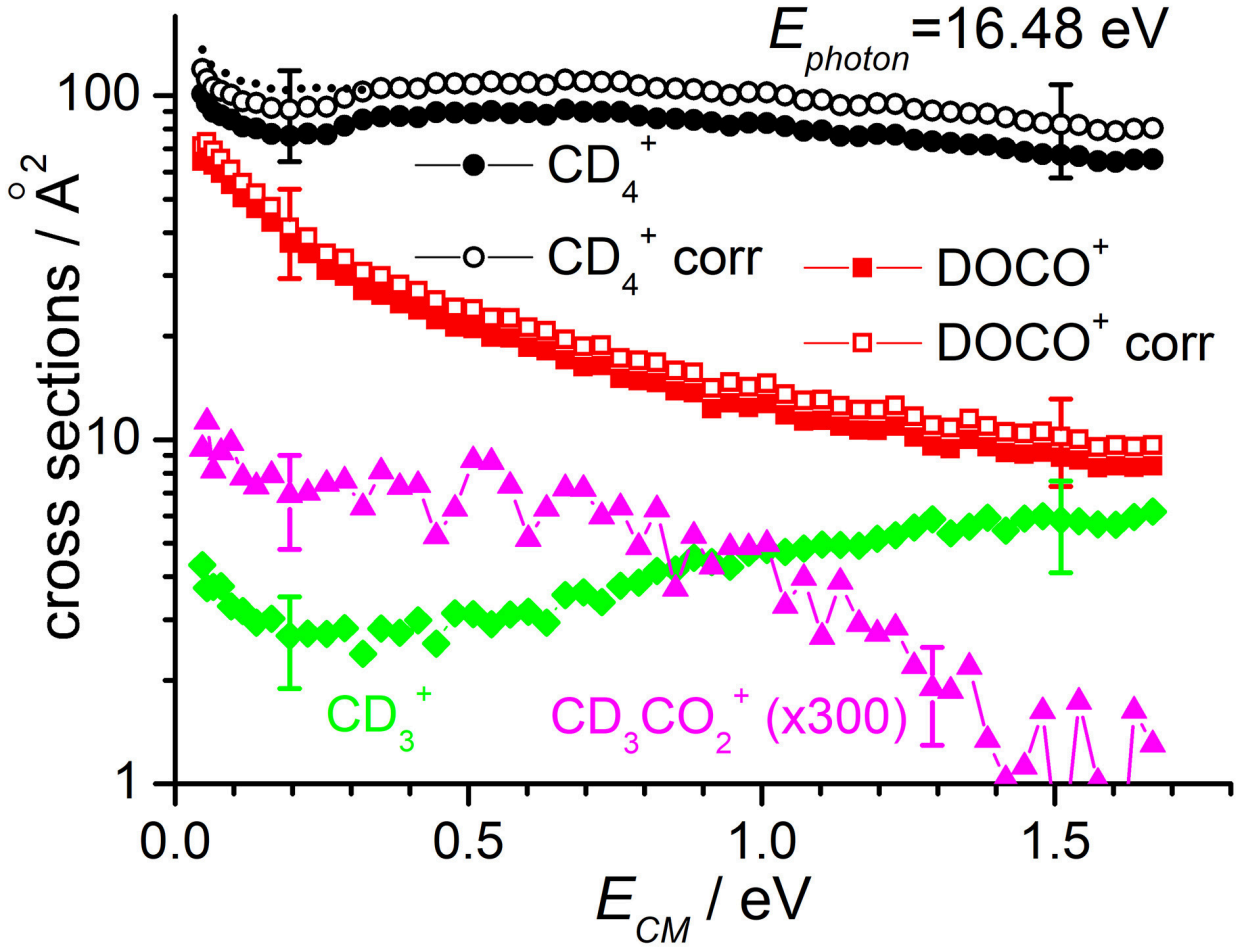
Reactive cross sections for products ${\text{CD}}_{4}^{+}$ (uncorrected, black circles), DOCO^+^ (uncorrected, red squares), ${\text{CD}}_{3}^{+}$ (green diamonds), and CD_3_${\text{CO}}_{2}^{+}$ (magenta triangles) measured as a function of the collision energy (*E*_*CM*_) in the DC mode at a photon energy *E*_*phot*_ = 16.48 eV. The open black circles and open red squares are the ${\text{CD}}_{4}^{+}$ and DOCO^+^ cross sections corrected to include the contribution of secondary reactions leading to ${\text{CD}}_{5}^{+}$ (data not shown). The dotted line represents the ${\text{CD}}_{4}^{+}$ cross sections corrected for the instrumental effect due to decreased collection efficiency at low *E*_*CM*_ (see text for details). Error bars on all the data are about 30%: for the sake of clarity only two error bars are reported, at arbitrarily chosen low and high collision energy values.

Cross sections for products ${\text{CD}}_{4}^{+}$, DOCO^+^ and ${\text{CD}}_{3}^{+}$ as well as ${\text{CD}}_{5}^{+}$ (from secondary reactions of the primary ${\text{CD}}_{4}^{+}$ and DOCO^+^ products) measured as a function of the collision energy when the reagent ${\text{CO}}_{2}^{+}$ ion is in its ground vibrational state (i.e., at *E*_*phot*_ = 13.78 eV) are reported in Figure 2, while results for *E*_*phot*_ = 16.48 eV are shown in Figure 3. The cross section for ${\text{CD}}_{5}^{+}$ was measured to correct the absolute value of the cross section for reactions (1) and (2) due to product ion losses via the highly efficient secondary reactions operative at the deuterated methane pressures used:

(6) $${\text{CD}}_{\text{4}}^{\text{+}}{\text{+CD}}_{\text{4}}\to {\text{CD}}_{\text{5}}^{\text{+}}{\text{+CD}}_{\text{3}}$$

(7) $${\text{DOCO}}^{\text{+}}{\text{+CH}}_{\text{4}}\to {\text{CD}}_{\text{5}}^{\text{+}}{\text{+CO}}_{\text{2}}$$

with *k* = 1.1 × 10^−9^ cm^3^·molecule^−1^·sec^−1^ for reaction (6) (Anicich, 2003) and *k* = 7.2 × 10^−10^ cm^3^·molecule^−1^·sec^−1^ for reaction (7) (Anicich, 2003). The measured ${\text{CD}}_{5}^{+}$ yields have been redistributed among the ${\text{CD}}_{4}^{+}$ and DOCO^+^ products on the basis of the ${\text{CD}}_{4}^{+}$/DOCO^+^ yield ratio (that changes with collision energy from ~2 at low energies up to ~7 at high energies) and of the different rate coefficients for reactions (6) and (7). In Figures 2, 3 the uncorrected and corrected cross sections for ${\text{CD}}_{4}^{+}$ are labeled as “${\text{CD}}_{4}^{+}$” and “${\text{CD}}_{4}^{+}$ corr” respectively, and the same notation is used for DOCO^+^.

The sudden increase in the cross section shown for ${\text{CD}}_{4}^{+}$ and ${\text{CD}}_{5}^{+}$ products at *E*_*CM*_ ~ 0.3 eV (clearly visible in the data of Figure 2, but also present in the data of Figure 3) is an instrumental effect due to a decrease in the collection efficiency for “slow” products. In particular, when product ions are produced at very low velocities in the lab frame, they have a chance to move backwards in the 1st octopole (O1) in the opposite direction from the parent ions, and they then face the last electrode before this octopole, L3, which is set to a potential of −0.4 V. As these ions are produced in O1, their fate depends on their initial kinetic energy and the relative values of the O1 and L3 potentials. At high collision energies, the mean potential of O1 is very low (negative values) and all product ions going back in O1 are reflected on L3 and later detected. At low collision energies, the O1 potential can be higher than that of L3, and backward product ions can be lost, accounting for the step in the product yield observed below collision energies of ~0.3 eV. To correct for such effect, we have rescaled the data measured at *E*_*CM*_ ≤ 0.3 eV by a fixed multiplication factor, chosen equal to 1.12 to match the data measured at *E*_*CM*_ < 0.3 eV with those measured at higher collision energy. Implicit in this way of rescaling data is the assumption that the number of product ions lost at low collision energy is independent on the collision energy. The corrected data (reported only for ${\text{CD}}_{4}^{+}$ cross sections corrected for the presence of secondary reactions leading to ${\text{CD}}_{5}^{+}$) are shown as dashed lines in Figures 2, 3.

According to the above-mentioned thermochemistry, the formation of ${\text{CD}}_{3}^{+}$ via either (3) or (4) is endothermic and the cross-section for its formation, when ${\text{CO}}_{2}^{+}$ is generated with no vibrational excitation (see green diamonds in Figure 2), shows the expected threshold behavior, with an appearance energy compatible with the endothermicity. Above threshold, the cross section increases accordingly with collision energy. The small amount of signal observed below the threshold is an artifact due to the tail of the very intense mass peak at 20 *m/z* (${\text{CD}}_{4}^{+}$). Data measured at *E*_*phot*_ = 16.48 eV show non-negligible cross sections even at low *E*_*CM*_, thus indicating that vibrational excitation of the ${\text{CO}}_{2}^{+}$ cation can promote the endothermic channel.

At *E*_*phot*_ = 13.78 eV and at the CD_4_ pressure used, the yield of CD_3_${\text{CO}}_{2}^{+}$ was below the detection limit, while at *E*_*phot*_ = 16.48 eV it was possible to measure a cross section for this very minor channel (data for CD_3_${\text{CO}}_{2}^{+}$ in Figure 3 are multiplied by 300 to be able to show them in the same scale of the other products).

In Table 2 results at the two photon energies are summarized by reporting the branching ratios (BRs) for the observed product channels and the energy-dependent rate constants. BR for the *i*-th channel have been calculated from the absolute cross sections according to the expression:

$$BR\left(i\right)=\frac{\sigma_{i}}{\sum \sigma_{i}}$$

**Figure 4:**
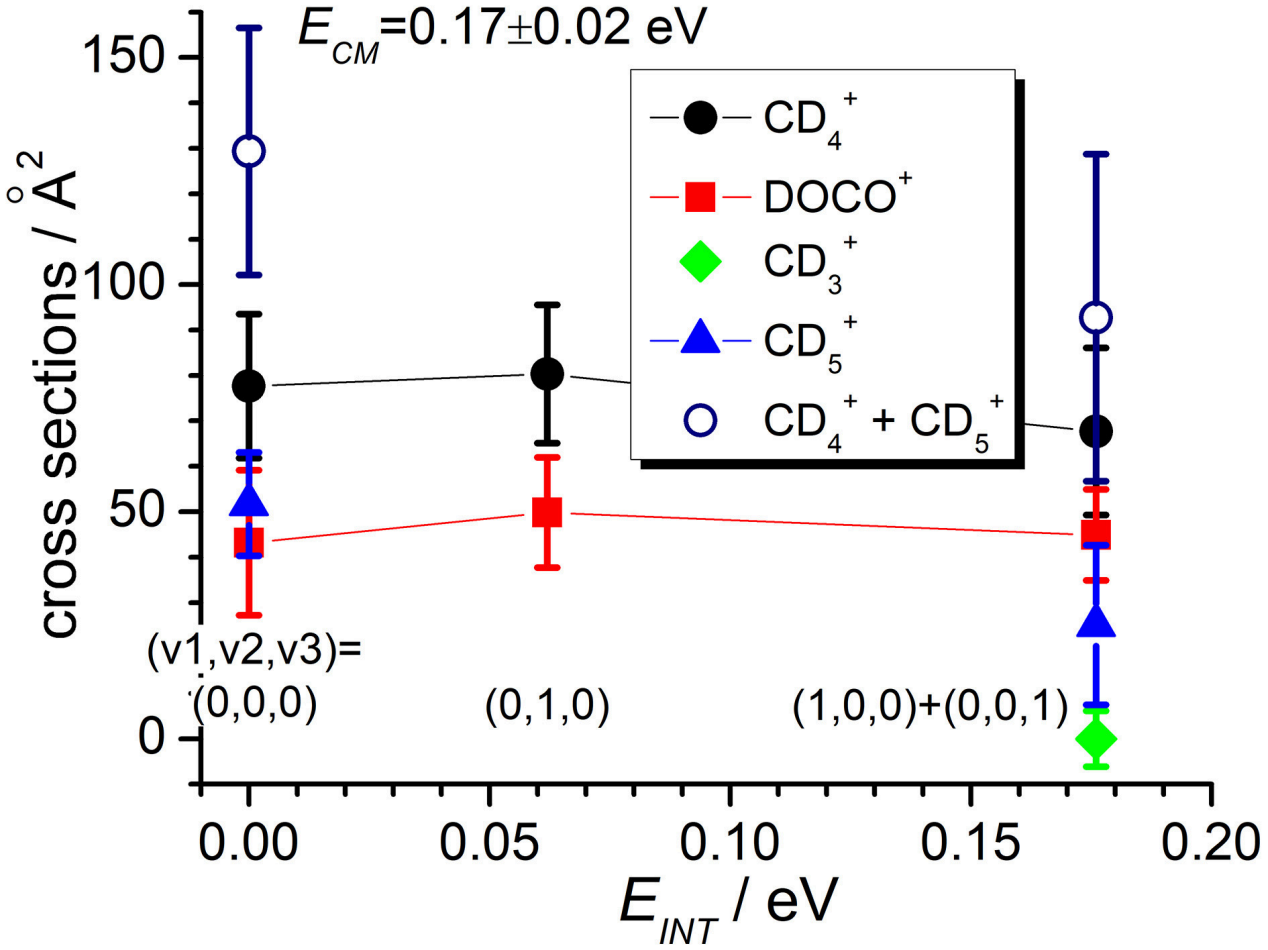
Reactive cross sections for products ${\text{CD}}_{4}^{+}$ (uncorrected, black circles), DOCO^+^ (red squares), ${\text{CD}}_{3}^{+}$ (green diamonds), and ${\text{CD}}_{5}^{+}$ (blue triangles) measured as a function of the vibrational excitation of the ${\text{CO}}_{2}^{+}$ reagent (*E*_*INT*_) at a fixed collision energy *E*_*CM*_ = 0.17 ± 0.02 eV. The open circles are the ${\text{CD}}_{4}^{+}$ cross sections corrected for the contribution of secondary reactions leading to ${\text{CD}}_{5}^{+}$. Lines connecting points are only a guide for the eyes.

The energy-dependent total rate constants *k*_*tot*_(*E*_*ave*_) have been estimated using the expression *k*_*tot*_(*E*_*ave*_) = 〈V〉 · σ_tot_, were σ_tot_ is the total reaction cross section (i.e., ∑σ_*i*_), as measured in this work (see data in Figures 3, 4) and < v> is the average relative velocity that can be estimated from the collision energy *E*_*CM*_ (see Ervin and Armentrout, 1985 and Nicolas et al., 2002 for a more detailed treatment). While the total rate constants do not change (within the error bars) when increasing the amount of internal excitation of the ${\text{CO}}_{2}^{+}$ cation (compare the values at different photon energies but same *E*_*ave*_), a slight change in the BRs is observed when increasing the collision energy, which favors the production of ${\text{CD}}_{4}^{+}$ (and ${\text{CD}}_{3}^{+}$) over that of DOCO^+^.

**Table 2:** Energy-dependent rate constants and branching ratios (BRs) for the title reaction measured in the DC mode at two different values of photon energies (*E*_*phot*_) and average collision energies (*E*_*ave*_).

	***E_phot_* = 13.78 eV**		***E_phot_* = 16.48 eV**	
	***E_***ave***_* = 0.1 eV**	***E_***ave***_* = 1.9 eV**	***E_***ave***_* = 0.1 eV**	***E_***ave***_* = 1.7 eV**
*k_*tot*_*(*E_*ave*_*)^a^	(1.8 ± 0.5) × 10^−9^	(4.9 ± 1.5) × 10^−9^	(1.8 ± 0.5) × 10^−9^	(4.7 ± 1.4) × 10^−9^
	**Branching ratios (BRs)**			
${\text{CD}}_{4}^{+}$	0.63 ± 0.06	0.82 ± 0.08	0.62 ± 0.06	0.84 ± 0.08
DOCO^+^	0.36 ± 0.04	0.08 ± 0.008	0.36 ± 0.036	0.10 ± 0.009
${\text{CD}}_{3}^{+}$	(8.9 ± 0.9) × 10^−3^	0.10 ± 0.01	(2.0 ± 0.2) × 10^−2^	(6.4 ± 0.6) × 10^−2^
CD_3_${\text{CO}}_{2}^{+}$	n.d.	n.d.	<10^−3^	<10^−4^

a *Total (i.e., summed over all the product channels) rate constant (in cm^3^·molecule^-1^·s^-1^) at the specified average collision energy E_ave_, estimated as detailed in the text*.

Interestingly, our results for the branching ratios (see Table 2) show reaction (1) to be the dominant channel at all the explored collision energies, in net disagreement with some of the existing values for the branching ratios (see Table 1). As already mentioned, in fact, in the literature there is a spread of the branching ratios for reaction (1) and (2) ranging from 1:0 (Durup-Ferguson et al., 1983) to 0.6:0.4 (Smith et al., 1978), to 0.5:0.5 (Copp et al., 1982), to 0.28:0.72 (Tsuji et al., 1994) and 0.25:0.75 (Huntress et al., 1980), and finally to 0:1 (Harrison and Myher, 1967; Rakshit and Warneck, 1980). We provide here some explanations for the differences observed between our experiment and previous ones:

1. In addition to working with CD_4_, in our experiment we perform a mass selection of the parent ion before reaction. In this way, we eliminate the contribution due to the ^13^${\text{CO}}_{2}^{+}$ parent ion that appears at the same *m/z* as the HOCO^+^ product (from residual not fully deuterated methane) and represents ~1% of the parent ion intensity. Additionally, our experimental procedure consists in measuring both parent and product ion yields first with the target gas in the collision cell, secondly with the target gas in the chamber. In this way, we correct for any contribution of “impurities” coming from the source at the same mass as the product (namely ^13^${\text{CO}}_{2}^{+}$/HOCO^+^). In one of the earlier papers (Tsuji et al., 1994), the experiment is performed without parent ion mass selection, and no correction for ^13^${\text{CO}}_{2}^{+}$ is mentioned. Hence the claimed HOCO^+^ branching ratio (0.72) is most likely overestimated. In Durup-Ferguson et al. (1983), the parent ion mass selection is performed, although no indication is given about the mass resolution. In Rakshit and Warneck (1980), a mixing of ${\text{CO}}_{2}^{+}$, CO_2_${\text{CO}}_{2}^{+}$ and H_2_O^+^ parent ions are used. The values from Huntress et al. (1980) are given without any experimental details, for which a reference is given to an earlier paper (Huntress, 1977) where the ICR set-up is described. However, the earlier paper does not contain data for the title reaction, and it is impossible to infer whether and how the ^13^${\text{CO}}_{2}^{+}$ contribution was taken into account.

2. In our experiment, we keep the target gas pressure as low as possible to limit the number of secondary reactions. Some of the earlier works (Harrison and Myher, 1967; Kasper and Franklin, 1972; Tsuji et al., 1994) have a pressure in the reaction cell higher than ours by a factor 30-50, with about the same cell length. Such differences may lead to underestimating the BR for ${\text{CH}}_{4}^{+}$ if secondary reactions are not adequately accounted for (as done in our study).

3. In our experiment, we use a pure target gas, while some of the earlier works perform mass spectrometry studies in mixtures of gases. If some CO_2_ is present in the region where ${\text{CH}}_{4}^{+}$ products are generated by the ${\text{CO}}_{2}^{+}$ + CH_4_ reaction, the ${\text{CH}}_{4}^{+}$ will be easily consumed by the efficient reaction ${\text{CH}}_{4}^{+}$ + CO_2_ → HOCO^+^ + CH_3_ (*k* = 1.2 × 10^−9^ cm^3^·molecule^−1^·s^−1^) that will produce HOCO^+^, leading to a negative bias in the ${\text{CH}}_{4}^{+}$/HOCO^+^ ratio. This is an issue in Rakshit and Warneck (1980) as highlighted by Copp et al. (1982), where CO_2_ is present in the reaction cell, as well as in Ryan and Harland (1974), where mixing of CH4 and CO_2_ occurs in the reaction region, in Tsuji et al. (1994), Harrison and Myher (1967) and Kasper and Franklin (1972).

4. In our experiments we mass select ionic products. Hence we can directly give BRs among different channels. In some of the flow/drift tube experiments, the reaction rate constants are measured by observing the decline of the primary ion signal upon addition of the neutral gas. For instance, in Durup-Ferguson et al. (1983) no mention is made about the mass detection of products, as if the authors have assumed the exclusive formation of ${\text{CH}}_{4}^{+}$ via non-dissociative CT, not considering the possibility that HOCO^+^ might be produced.

### Results in the TPEPICO Mode

**Figure 5:**
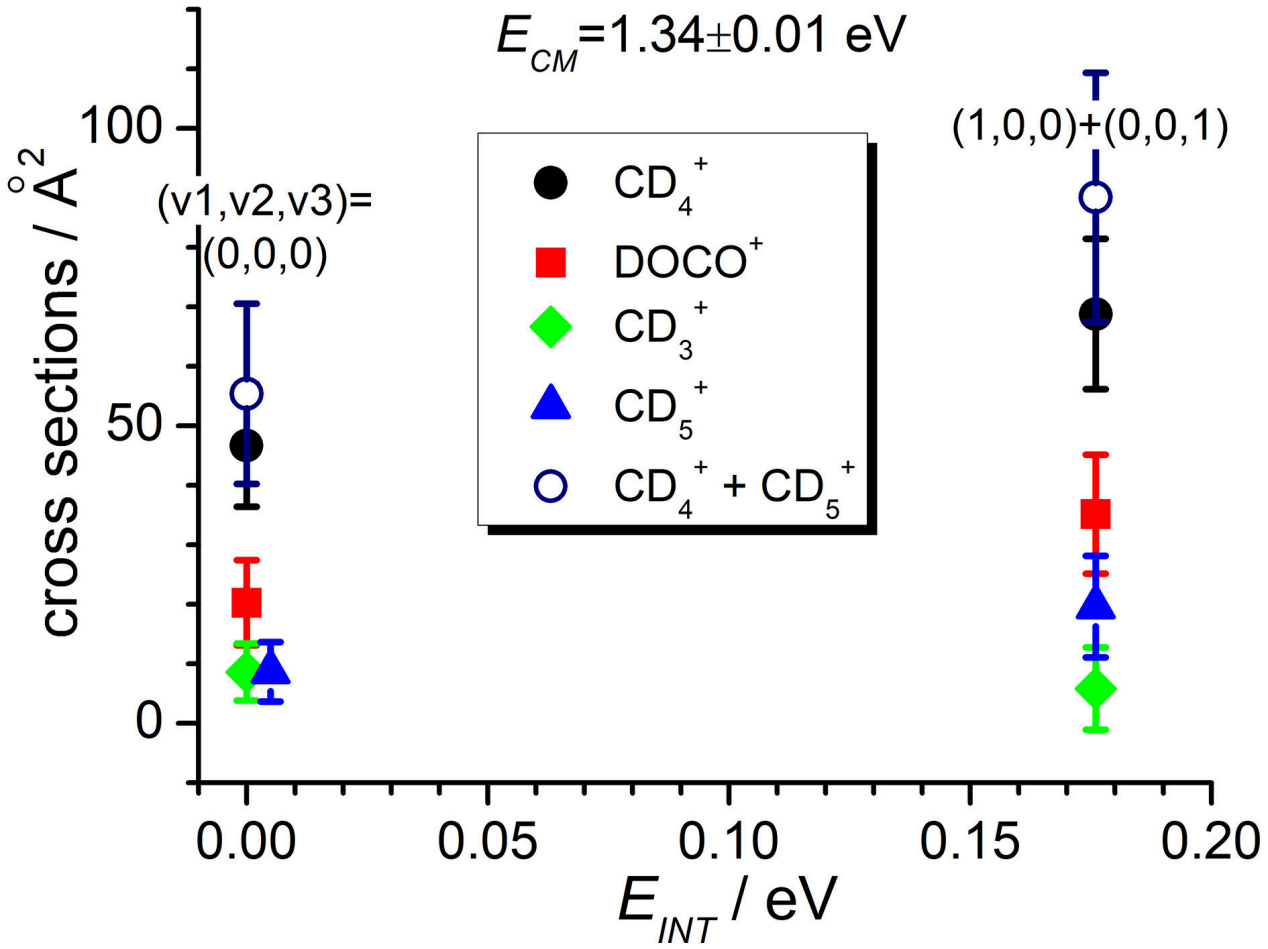
Reactive cross sections for products ${\text{CD}}_{4}^{+}$ (uncorrected, black circles), DOCO^+^ (red squares), ${\text{CD}}_{3}^{+}$ (green diamonds), and ${\text{CD}}_{5}^{+}$ (blue triangles) measured as a function of the vibrational excitation of the ${\text{CO}}_{2}^{+}$ reagent (*E*_*INT*_) at a fixed collision energy *E*_*CM*_ = 1.34 ± 0.01 eV. The open circles are the ${\text{CD}}_{4}^{+}$ cross sections corrected for the contribution of secondary reactions leading to ${\text{CD}}_{5}^{+}$.

In the TPEPICO mode, we recorded cross sections for the reaction of ${\text{CO}}_{2}^{+}$ ions in the (0,0,0) ground state and in two vibrationally excited states: (0,1,0) with one quantum of bending vibration and [(1,0,0) + (0,0,1)] corresponding to a combination of the symmetric and antisymmetric stretching vibration. Cross sections were measured for products ${\text{CD}}_{4}^{+}$, DOCO^+^ and ${\text{CD}}_{3}^{+}$ as well as ${\text{CD}}_{5}^{+}$ (from secondary reactions of the primary ${\text{CD}}_{4}^{+}$ products, see above). The ion yield for product CD_3_${\text{CO}}_{2}^{+}$ was too low to be detectable in coincidence. For most of the other products, reactive cross sections were measured at two different collision energies *E*_*CM*_ = 0.17 ± 0.02 eV and 1.34 ± 0.01 eV and results are shown in Figures 4, 5. Despite the larger uncertainties (due to the low S/N ratio in the coincidence mode) cross section measurements are consistent with results obtained in the DC mode. In particular, when converting cross sections reported in Figure 4 for ${\text{CO}}_{2}^{+}$ at low collision energy and in the (0,0,0) ground state we obtain the following BRs: ${\text{CD}}_{4}^{+}$ (0.75 ± 0.25), DOCO^+^ (0.25 ± 0.11) and ${\text{CD}}_{3}^{+}$ (0.00 ± 0.03), entirely consistent, within the error bars, with the data obtained in the DC mode (see Table 2, second column from the left).

In addition, the TPEPICO results confirm that cross sections for the exothermic CT (1) and deuterium-atom-transfer (2) channels change very little with increasing vibrational excitation, and the increase is more evident in data taken at high collision energy (Figure 5). Cross sections for the endothermic channel leading to ${\text{CD}}_{3}^{+}$ increase with collision energy but they do not seem to depend strongly on the internal excitation of the ionic parent, at least for the low internal excitations here explored (i.e., maximum one quantum of vibrational excitation).

The observed small dependence of reactions (1) and (2) from ${\text{CO}}_{2}^{+}$ vibrational excitation requires some consideration. First of all, we note that our result is in agreement with a previous study (Durup-Ferguson et al., 1983) in which a drift tube technique is used, and the dependence of the reaction rate constant on the internal energy of the ions is examined by varying the mass of the buffer gas. Despite the limitations of the technique compared to our truly state-selection method, in Durup-Ferguson et al. (1983) the non-dissociative CT giving ${\text{CH}}_{4}^{+}$ plus CO_2_ is found to occur at near the collision rate and to have little energy dependence and no measurable vibrational dependence.

We also note that, differently from the CH_4_ case, vibrational excitation of ${\text{CO}}_{2}^{+}$ ions was found to enhance the CT reaction probability with O_2_ (Durup-Ferguson et al., 1983; Ferguson et al., 1992; Viggiano and Morris, 1996; Nicolas et al., 2002). To rationalize such results, it should be considered that the two systems present several differences:

- CT channels have different exothermicities (−1.17 eV for CH_4_ and −1.71 for O_2_).
- The CT rate constant is substantially larger for CH_4_ (*k* = 0.3–1 × 10^−9^ cm^3^·molecule^−1^·s^−1^, see values in our Table 1) than for O_2_ (*k* ~ 5 × 10^−11^ cm^3^·molecule^−1^·s^−1^).

The inefficiency of the CT with O_2_ is attributed to the non-resonant character of the reaction, i.e., to the fact that Franck-Condon factors for O_2_ ionization at the ionization potential of CO_2_ (13.78 eV) are close to zero (see for instance Wacks, 1964). On the other hand, it is known that for CH_4_ the Franck-Condon factors are low at the ionization threshold and increase reaching a maximum in the region 13.5–14.5 eV (see for instance Stockbauer and Inghram, 1971). Thus, while in the CH_4_ case CT can occur efficiently at long range via the direct mechanism previously described, for O_2_ it should involve the formation of a collision complex. As tentatively explained and demonstrated in Ferguson et al. (1992), in the O_2_ case a small amount in the stretching excitation of ${\text{CO}}_{2}^{+}$ can lead to an increase in the CT probability. Hence, CH_4_ and O_2_ are quite different reaction systems, and in the latter case, the effect of ${\text{CO}}_{2}^{+}$ excitation could be more pronounced than for a reaction already at the Langevin limit (as in CH_4_).

More generally, the dynamics of CT processes is regulated by crossings among entrance and exit potential energy surfaces. When such crossings are occurring at distances for which the probability of electron transfer from one adiabatic PES to the other is unfavorable, the CT cross section will be very small even for exothermic processes (according to the Landau-Zener model for CT probability). The fact that increasing the vibrational excitation of the cation does not increase the CT cross section can be related to the fact that the crossing probability does not change much when increasing the vibrational excitation of ${\text{CO}}_{2}^{+}$, even though the exothermicity increases. Unfortunately, modeling the dynamics occurring on a ${\text{CO}}_{2}^{+}$- CH_4_ multidimensional potential energy surface which includes vibrational excitation of CH_4_ is not an easy task, and it is beyond the scope of our paper. We hope that our results will stimulate theoreticians and experts in *ab-initio* calculations to use this system as a test bench for theory.

## Conclusions

The reactivity of ${\text{CO}}_{2}^{+}$ with deuterated methane has been investigated experimentally by guided ion beam mass spectrometric techniques by changing either the kinetic energy of ${\text{CO}}_{2}^{+}$ or its vibrational excitation (using synchrotron radiation in the VUV energy range to produce vibrationally excited reagent ions). The main products are ${\text{CD}}_{4}^{+}$, DOCO^+^, and ${\text{CD}}_{3}^{+}$ and reactivity is found to depend on the reagent collision energy, but not so much on the vibrational excitation of ${\text{CO}}_{2}^{+}$.

An interesting issue is whether reaction rates and dynamics change or remain the same when CD_4_ is replaced by CH_4_. We do not expect charge transfer cross sections to be affected by a strong kinetic isotope effect (KIE). On the other hand, one can foresee a kinetic isotope effect in the H/D atom transfer channel leading to HOCO^+^/DOCO^+^. In particular, according to the semi-classical theory of primary KIE a normal effect (i.e., k_H_/k_D_ > 1) is expected in the transfer of an H/D atom due to the vibrational zero-point energy differences for each of the vibrational modes of the reactants and transition state. Despite the limited mass resolution in our experimental set-up, we have managed to perform some tests using CH_4_ in the reaction cell and measuring BRs and cross sections at a fixed collision energy of 0.11 eV in the DC mode at a photon energy *E*_*phot*_ = 13.78 eV (i.e., same conditions of Figure 2). Products are observed at *m/z* values corresponding to ${\text{CH}}_{4}^{+}$, ${\text{CH}}_{5}^{+}$, and HOCO^+^. By correcting for secondary reactions (as detailed in the text) we obtain a BR of 0.74:0.26 = CH4^+^: HOCO^+^ and a total cross section of 199 (±30%) Å^2^, corresponding to an energy-dependent rate constant of (2.68 ± 0.8) × 10^−9^ cm^3^·molecule^−1^·s^−1^ to be compared with the CD_4_ value of (1.8 ± 0.5) × 10^−9^ at similar *E*_*ave*_ (see our results in Table 2) and the BR of 0.63:0.36 = ${\text{CD}}_{4}^{+}$: DOCO^+^. This means a positive KIE k_H_/k_D_ = 1.5(±0.6). On the other hand, our results show that when CD_4_ is replaced by the lighter isotopolog, the CT is more favored than the H atom transfer. This effect can be explained assuming that the different vibrational spacings in CD_4_/CH_4_ might change the Franck-Condon factors and the efficiencies of non-adiabatic transition probability among the entrance and exit potential energy surfaces. We note that in a similar reacting system CN^+^ plus CH_4_/CD_4_ a KIE in the total rate coefficient similar to the one observed in our case has been reported, namely k_H_/k_D_ = 1.55 (±0.66) (Raksit et al., 1984).

To put our results into the context of plasma chemistry used for the conversion of CO_2_ in carbon-neutral fuels (Snoeckx et al., 2013; Snoeckx and Bogaerts, 2017), both the products (${\text{CH}}_{4}^{+}$ and HOCO^+^) of the reaction of ${\text{CO}}_{2}^{+}$ with CH_4_ eventually lead to the production of ${\text{CH}}_{5}^{+}$ and CH_3_, as shown in the following scheme:


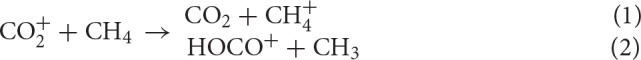


(8) $${\text{CH}}_{\text{4}}^{\text{+}}\text{+ }{\text{CH}}_{\text{4}}\to {\text{CH}}_{\text{5}}^{\text{+}}{\text{+ CH}}_{\text{3}}$$

(9) $${\text{CH}}_{\text{4}}^{\text{+}}\text{+ }{\text{CO}}_{\text{2}}\to {\text{HOCO}}^{\text{+}}\text{+ }{\text{CH}}_{\text{3}}$$

(10) $${\text{HOCO}}^{\text{+}}\text{+ }{\text{CH}}_{\text{4}}\to {\text{CH}}_{\text{5}}^{\text{+}}\text{+ }{\text{CO}}_{\text{2}}$$

Thus the energy initially used to ionize CO_2_ is transferred to CH_4_ to form ${\text{CH}}_{4}^{+}$, ${\text{CH}}_{5}^{+}$ and CH_3_. Only processes (2) and (9) lead to HOCO^+^ that, in addition to react with CH_4_, giving back CO_2_, can also recombine with electrons to yield CO plus OH.

## Data Availability

The datasets generated for this study are available on request to the corresponding author.

## Author Contributions

DA, CA, and PT contributed conception and design of the study. CA, CR, and JŽ planned and developed the experimental set-up. DA, CR, CA, AL, JŽ, MP, and CS contributed to data acquisition, data analysis, and interpretation of results. DA wrote the first draft of the manuscript. DA, PT, and CA wrote sections of the manuscript. All authors contributed to manuscript revision, read and approved the submitted version.

### Conflict of Interest Statement

The authors declare that the research was conducted in the absence of any commercial or financial relationships that could be construed as a potential conflict of interest.

## Footnotes

^1^*NIST Chemistry WebBook, NIST Standard Reference Database Number 69* (2017), eds. P.J. Linstrom, and W.G. Mallard (Gaithersburg, MD: National Institute of Standards and Technology), 20899.
